# The induction of bone formation by 3D-printed PLGA microsphere scaffolds in a calvarial orthotopic mouse model: a pilot study

**DOI:** 10.3389/fbioe.2024.1425469

**Published:** 2024-10-25

**Authors:** Roland M. Klar, James C. Cox, Claire J. Houchen, Naren Raja, Houssam Bouloussa, Stefan Lohfeld

**Affiliations:** ^1^ Department of Oral and Craniofacial Sciences, University of Missouri-Kansas City, School of Dentistry, Kansas City, MO, United States; ^2^ Department of Orthopedic Surgery, University of Missouri-Kansas City, School of Medicine, Kansas City, MO, United States

**Keywords:** PLGA, microsphere scaffolds, orthotopic site, bone formation, 3D bioplotter, tissue engineering, regenerative medicine, bioengineering

## Abstract

Polymeric biodegradable microspheres are readily utilized to support targeted drug delivery for various diseases clinically. 3D printed tissue engineering scaffolds from polymer filaments with embedded microspheres or nanoparticles, as well as bulk microsphere scaffolds, have been investigated for regenerative medicine and tissue engineering. However, 3D printed scaffolds consisting only of a homogenous microsphere size with an optimized architecture that includes a unique micro- and macroporosity, have been challenging to produce and hence, have not been assessed in the literature yet. Utilizing our recently established 3D-MultiCompositional Microsphere-Adaptive Printing (3D-McMap) method, the present study evaluated the effectiveness of 3D-printed poly (lactic-co-glycolic acid) (PLGA) microsphere scaffolds, consisting of microsphere sizes 50, 100, or 200 μm, on the induction of bone formation when implanted in the calvarial murine regeneration model. Our results showed that PLGA microsphere scaffolds possess unique properties that support bone regeneration by supporting osteoconduction and stimulating, in our opinion, true spontaneous osteoinduction. The study demonstrated that PLGA microsphere-based scaffolds support bone growth in the absence of additional growth factors and promote osteogenesis primarily via their unique geometric configuration. The larger the microspheres were, the greater *de novo* bone formation was. This proves that bone tissue engineering scaffolds 3D printed from microspheres, enabled by the 3D-McMap method, are superior over bulk material printed scaffolds, as they possess the unique capability of spontaneous induction of new bone formation. With the addition of encapsulated modulatory bone-forming biomolecules they can substantially improve the spatiotemporal control of tissue morphogenesis, potentially leading to new innovative clinical tissue repair therapies that regenerate bone in large defects correctly and fully.

## 1 Introduction

While bone can robustly maintain itself throughout a person’s lifespan and possesses self-regenerative properties when fractured or lightly damaged (Keating et al., 2005; Schmitz and Hollinger, 1986), large bone defects, termed critical-sized defects, are incapable of healing spontaneously (Fong et al., 2003; Schmitz and Hollinger, 1986). The autogenous bone graft remains the clinical gold standard for repairing critical-size defects (Burchardt, 1983; Cypher and Grossman, 1996; Zhou et al., 2021). While more than 250 synthetic and biological biomaterials exist, none can fully recapitulate the unique reaction stimulated by the autogenous bone graft (Baldwin et al., 2019; Nandi et al., 2010; Zhou et al., 2021). Though ceramics and other synthetic bone substitute scaffolds, demineralized bone matrix pastes, or implantation of inorganic material such as metal-based implants have shown some benefit in the fields of dentistry and endoprosthetics (Kim et al., 2007; Najeeb et al., 2016), these methods are still imperfect and require substantial improvements to meet clinical standards, as achieved by the autologous bone grafts (An et al., 1995; Eastlund, 2006; Journeaux et al., 1999; Lerner et al., 2009; Pawelke et al., 2023). Therefore, new bioengineering approaches and technologies are desperately required to meet a growing call for reliable methods that adequately recapitulate the process of *de novo* bone growth in critical-sized bone defects. Such methods must be promising for translation into the clinic by seamlessly and efficiently regenerating a patient’s bone. One such bioengineering technique is the utilization of 3-dimensional (3D) printed microsphere scaffolds to create superior temporospatial structures, thus possibly replicating the successful induction of bone formation (Abdelaziz et al., 2023; Gupta et al., 2017; Klar et al., 2024).

Microspheres are 1–1,000 μm spheroid structures made of either organic or inorganic material such as albumin, gelatin, starch, polyanhydride, dextran, polylactide, polyglycolide, chitosan, polyphosphoazene and/or oils (Gupta et al., 2017; Karadağ and Omarova, 2024). Importantly, microspheres can contain encapsulated drugs or other bioactive molecules, allowing for improved drug release kinetics and controlled reactivity. Microspheres are an excellent option for 3D-printed scaffolds because they offer great flexibility. Microsphere material, microsphere size, space between microspheres, the strength of microsphere-microsphere bonds, and the presence/absence of bioactive molecules can all be modulated to suit the biological context into which the scaffold will be placed (Gupta et al., 2017). Current 3D printing techniques have difficulties effectively printing microspheres directly, significantly limiting the targeted deposition within a 3D structure. To alleviate this issue biomedical science has concentrated more on nanoparticle based bioinks as not only is the manufacture easier but 3D bioprinting processes are also simpler with less and smaller hard matter in the bioink. Various studies using nanoparticles have shown similar cellular responses to that of microspheres (Salahshour et al., 2024). However, even so it still remains unresolved if a true printed microsphere-based scaffold without any growth factors has at all any reaction in stimulating a tissue wide response, as most pure polymer based scaffolds without the addition of either microspheres or nanoparticles, and including growth factors, do not regenerate tissues *in vivo* (Gupta et al., 2017; Salahshour et al., 2024). Our literature research going back to as early as the 1970s, when the concept of microparticles was theorised as a benefical possibily for medicine, tissue regeneration, and biomedicine, up to 2024 has revealed no information on whether 3D printed polymeric microsphere-based scaffolds even possess any tissue stimulative capabilites *in vivo* (Gupta et al., 2017; Klar et al., 2024). Generally, when microspheres are used in scaffolds, they are entirely buried in another phase, losing the collective advantage of their shape, properties, and high surface-to-volume ratio, and are available in relatively small quantities compared to the volume of the scaffold versus nanoparticles (Corcione et al., 2019; Legemate et al., 2016; Tarafder et al., 2016; Salahshour et al., 2024). However, both nanoparticles and microspheres often require the embedding material to degrade first before they can exert an effect. The extrusion processes often used for printing scaffolds typically cannot create a microporosity beneficial for cell attachment and proliferation (Dang et al., 2019; Hing et al., 2005; Visscher et al., 2018) within the printed struts. Hence, generating a 3D printed scaffold for biomedical applications consisting solely of microspheres should provide the highest control over releasing substances and with specific intrinsic geometrical configurations that are known to be essential for support cyto-differentiation (Ripamonti et al., 2010). Non-3D printed microsphere-based scaffolds have shown some success in partially stimulating bone formation *in vivo* when growth factors, such as platelet-derived growth factor, vascular endothelial growth factor, and bone morphogenetic protein-2, are encapsulated into the microspheres (Dormer et al., 2013; Fahimipour et al., 2017; Kempen et al., 2008). The same has yet to be shown to be true for 3D-printed microsphere-based scaffolds.

Since we have recently overcome the issue of manufacturing 3D-printed microsphere-based scaffolds utilizing our 3D multi-composite microsphere adaptive printing (3D-McMap) guidelines to generate evenly layered printed scaffolds consisting purely of microspheres (Klar et al., 2024), the present study aimed to investigate the influence of microspheres and different microsphere size on the bone conduction capabilities of 3D printed poly (lactic-co-glycolic acid) (PLGA) microsphere scaffolds, which could provide new and superior biomedical innovative solutions for regenerating large tissue defects. For this purpose, the scaffolds were implanted into critical-size cranial defects in a murine model.

## 2 Materials and methods

### 2.1 Microsphere scaffold manufacture

#### 2.1.1 Microsphere production (50 μm, 100 µm, 200 µm)

Microspheres with an average diameter of 50, 100, and 200 µm were produced from poly (lactic-co-glycolic acid) (PLGA; PURASORB PDLG 5004, Corbion, Amsterdam, Netherlands) crystals, using a Büchi Encapsulator B-390 (Büchi Labortechnik, Flawil, Switzerland). Briefly, to generate a microsphere size of 50, 100, and 200 μm, an inner core nozzle with 80, 120, or 200 µm diameter was respectively used, whereas for the outer shell nozzle 300 µm diameter, for all microsphere sizes was mounted on the microsphere manufacturing unit of the Encapsulator. The core fluid consisted of 4% (for the 50 µm microspheres only) and 5% (w/v) PLGA dissolved in dichloromethane (DCM, Thermo Fisher Scientific, Waltham, MA, United States). The shell fluid consisted of 0.33% (v/v) poly (vinyl alcohol) (PVA, Polyscience Inc., Warrington, PA, United States) in water. The nozzle assembly was immersed in a 0.33% PVA solution, as previously published (Klar et al., 2024), that was agitated using an Isotemp stirrer platform (Thermo Fisher Scientific) set at 220 rpm to prevent generated microspheres from lumping. Flow rates through the nozzle assembly, using syringe pumps (KD Scientific, Holliston, MA, United States), including the vibration unit’s frequency (Hz) to generate 50, 100, and 200 µm PLGA microsphere were set as follows.

−50 µm: 5 mL/min (shell fluid), 0.5 mL/min (core fluid) and 1800 Hz
−100 µm: 4.5 mL/min (shell fluid), 1.5 mL/min (core fluid) and 1,600 Hz
−200 µm: 4 mL/min (shell fluid), 2 mL/min (core fluid) and 1,000 Hz

The amplitude value was set at six for all cases. These parameters were found following the guidelines for the Büchi Encapsulator to generate microspheres in the desired size ranges. After microsphere production, the collection solution was stirred for another 8 h to allow for DCM evaporation and microsphere hardening. The microspheres were then thoroughly washed in distilled water, extracted, and lyophilized in a FreeZone 4.5PLus lyophilizer (Labconco Corporation, Kansas City, MO, United States). The dried microspheres were stored at −20°C until further use.

#### 2.1.2 Microsphere quality control

Prior to being utilized in the 3D printing process, the microsphere batches were sifted to ensure that at least 98% of the used microspheres fell into their respective size ranges. As previously published (Klar et al., 2024), United States standard sieves (Anylia Scientific, Vernon Hills, IL, United States) with a mesh size of 60 and 45 µm (for 50 µm microspheres), 106 μm–90 µm (for 100 µm microspheres) and 212 μm and 190 µm (for 200 µm microspheres), were utilized to separate the desired microsphere size range. Since PLGA microspheres possess high electrostatic forces (Müller et al., 2003) and tend to stick to the sieve’s wall, sucrose (Thermo Fisher Scientific) was milled into particles (∼30 µm) and added at a ratio of 1:1 to the microspheres. Sifting was performed under agitation with a No. 1A Vibrator (Buffalo Dental Inc. Syosset, NY, United States). Sifted microspheres were washed in distilled water to eliminate sucrose and analyzed under a fluorescence microscope (Keyence BZ-X800, Keyence Corporation, Osaka, Osaka, Japan) to validate quality and sucrose removal. After the sucrose removal was confirmed by fluorescence microscopy, the washed microspheres were extracted, re-lyophilized, and stored at −20°C until further use.

#### 2.1.3 Bioink preparation

For the microspheres, a solution of 3% carboxymethyl cellulose (CMC, Sigma-Aldrich, St. Louis, MO, United States) in DI water was prepared and mixed with the microspheres to produce the biomaterial ink. The ratio of PLGA microspheres to CMC solution was 5:3 (w/w). This ratio was standardized for all microsphere sizes used in the experiment when extruding the bio-ink from a 16G syringe tip used during 3D printing. The biomaterial ink was loaded into a 1 mL Luer lock syringe (Thermo Fisher Scientific) utilizing a custom rig, as previously published (Klar et al., 2024), to fit into the standard 30 mL syringes typically utilized in the 3D-Bioplotter system.

#### 2.1.4 3D-McMap printing

**FIGURE 1:**
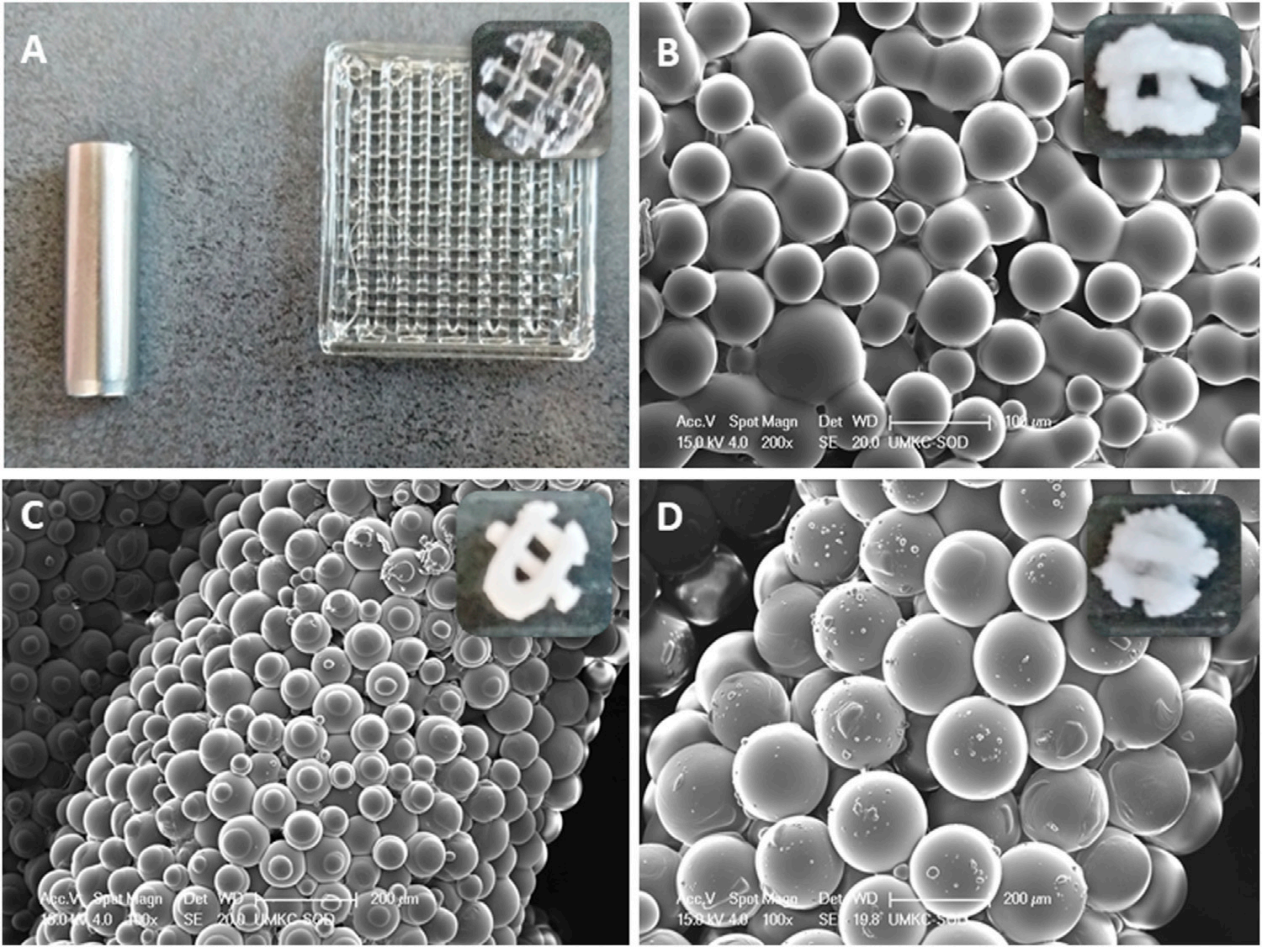
**(A)** A 3D-printed pure PLGA square scaffold from which, using a 5 mm biopsy punch scaffolds were obtained.; **(B)** ∼50 µm PLGA diameter microspheres in punched out scaffold; **(C)** ∼100 µm PLGA diameter microspheres in punched out scaffold; **(D)** ∼100 µm PLGA diameter microspheres in punched out scaffold.

The 3-dimensional multi-composite microsphere adaptive printing (3D-McMap) technique was used (Klar et al., 2024) in conjunction with an EnvisionTec 3D-Bioplotter system (EnvisionTec, Gladbeck, Germany). To obtain cylindrical scaffolds with a diameter of 5 mm x height 1.2 mm, scaffolds were punched out of printed 10 mm × 10 mm x 1.2 mm (width x breadth x height) pure PLGA (control) or PLGA microsphere-based rectangular scaffold, using a 5 mm biopsy punch (Integra Lifesciences, Plainsboro, NJ, United States) (Figure 1A). The three-dimensional PLGA structures were first designed in Perfactory Suite v3.1 (EnvisionTec) and then imported into the Visual Machines v2.1 software (EnvisionTec) of the 3D-Bioplotter. Following the recommendations of the printer’s manufacturer, layer thickness was set to approximately 80% of the inner diameter of the syringe tip used for printing. A 16 Gauge (16G; Nordson EFD, Nordson Corporation, Westlake, OH, United States) precision syringe tip was used, meaning the layer thickness for this model was set to 0.67 mm. A fill pattern consisting of continuous lines with a 1.7 mm distance between their centerlines was included in the printed shape. The contour was printed with a single line. Each layer was rotated by 90° to the previous one. Utilizing the built-in “Material parameters Tuning/Optimization” tool of the Visual Machines software, the starting printing parameters for pure PLGA (control) and PLGA microsphere scaffolds were set as follows.

1. Pure PLGA scaffold (Control)—extrusion printing was performed with a high-temperature print 22G head set to 150°C, needle offset was set to 0.67 mm, the starting extrusion pressure was 0.6 bar (stable—no pressure adaptations needed), printing speed was 8 mm/s, and printing stage temperature was 40°C (Figure 1A).
2. Extrusion printing of PLGA microsphere scaffolds was performed with a low-temperature print head set to four°C, needle offset set at 0.67 mm, extrusion pressure was 3 bars, printing speed was 1.5 mm/s, and printing stage temperature was 50°C (Figures 1B–D).

#### 2.1.5 Vapor sintering (dichloromethane)

Once dried, punched out 3D-printed PLGA microsphere scaffolds were sintered in dichloromethane vapor. As previously described (Klar et al., 2024), scaffolds were placed into a custom-designed vapor sintering chamber for proper dichloromethane gas penetration. PLGA scaffolds were sintered for exactly 165 s ± 0.5s.

### 2.2 Bone conduction experiments

#### 2.2.1 Animal implantation/surgery

A total of 24 clinically healthy adult TOPGAL mice (*Mus musculus*), 12 male and 12 female, with a mean weight of 43.7 ± 2.3 g, were selected from the TOPGAL colony of the University of the Missouri-Kansas City. TOPGAL mice were chosen for this study due to their high capacity to form new, thicker bone (DasGupta and Fuchs, 1999). Animals, after surgery, were housed separately to minimize wound trauma effects on the healing with food and water provided *ad libitum* under a 12-h light/dark cycle at 23°C–26°C. The research protocol (#2202) was approved by the University’s Institutional Animal Care and Use Committee (IACUC) and conducted according to the Guidelines for the care and use of Experimental Animals prepared by the University and in compliance with the Federal Code for Animal Use in Research, Education and Diagnosis in the United States (Underwood and Anthony, 2020). The calvarial model for tissue induction and morphogenesis was used in the surgical procedure. Animals were anesthetized with 5% isoflurane (Safco LLC, Skokie, IL, United States) using a custom chamber, and general anesthesia was maintained by 2%–3% isoflurane gas using a custom anesthesia mask. Under general anesthesia, the calvaria was exposed, and a single full-thickness defect of 5 mm in diameter was created in the calvarium with a craniotome under saline irrigation. The dura mater was left intact. One scaffold was positioned in the defect, and the wounds were closed with clip sutures (Safco LLC). Animals were injected postoperatively with a subcutaneous injection of 0.05–0.1 mg/kg buprenorphine (Safco LLC) and monitored daily.

#### 2.2.2 Tissue harvest, micro-computed tomography and histomorphometry

On day 60, animals were sacrificed. The head was surgically removed and fixed in 10% buffered formalin for 5 days, after which skin tissue was removed, leaving endocranial tissue intact and covering the defect area. Heads were rinsed in distilled water and subsequently stored in 70% alcohol prior to downstream analytical applications (Rahman et al., 2022).

Before histological and histomorphometric evaluation, calvarial specimens were imaged using microcomputed tomography (µCT) (Skyscan model 1,275 µCT; Bruker Corporation, Billerica, MA, United States) for qualitative and quantitative assessment. Using a custom Styrofoam holder, specimens were individually imaged at 17 µm isotropic resolution utilizing the following scan settings for all specimens: 55 V, 180 μA, 45 m exposure, 360° imaging, 0.2° rotation step, and six-frame averaging. The raw images from each scan were then reconstructed using NRecon software (v1.7.4.2; Bruker Corporation) using consistent greyscale settings and imported into Drishti volume exploration software (v3.0.0; https://github.com/nci/Drishti) for 3D rendering. The rendering settings were optimized for visualizing and assessing the bony defect, with the same setting applied to all scan volumes. To quantify the extent of new bone formation, each reconstructed multiplanar dataset was imported in DataViewer (v1.5.1.2; Bruker Corporation) for precise re-orientation to permit re-slicing of the dataset in the plane horizontal to the burr hole. Re-sliced data was then opened using CTAn software (Bruker Corporation). First, the area of the region of interest (ROI) for each mouse was calculated using square pixels and converted to square microns. The approximate area measurement from CTAn was confirmed by parallel length and width measurements performed in Drishti. Finally, to calculate the area for new bone formation, the ROI for each specimen was then compared to the area of a ‘perfect 5 mm diameter’ burr hole. The percentage of bone regeneration was then calculated using Equation 1:

$$100-\left(\frac{x}{\pi r²}\right)\;*\;100=\%\;bone\;regeneration$$ (1)

where x = the ROI area of each specimen and r = radius of the burr (2.5 mm).

Undecalcified specimens were treated according to the method of von Kóssa (Kóssa, 1901; Schneider, 2021). Specimens were processed in ascending grades of ethanol in an automatic tissue processor (Tissue Tech, V.I.P.; Miles Inc., Elkhart, United States) under pressure vacuum cycles. Specimens were infiltrated with an ascending concentration of methyl methacrylate (Sigma-Aldrich) and embedded in a fresh solution of the same resin. Specimens were first trimmed to obtain a total cross-sectional area of the specimens. Undecalcified specimens were then sectioned, thickness ∼5–6 μm, using an Epredia HM355S Automatic Section microtome (Thermo Fisher Scientific). The sections were then stained following the histological staining procedure of von Kóssa to determine new bone formation within the calvarial specimens. Sections were analyzed under a fluorescence microscope (Keyence BZ-X800, Keyence Corporation, Osaka, Japan), and images were taken for histological analysis.

To correlate the quantities generated from µCT scanning, histomorphometric analysis was also performed on the histologically stained sections. ImageJ (https://imagej.nih.gov/ij/) software was used to perform the Histomorphometric analysis. The ratio of the positive area to the total area of each section was calculated as a semi-quantitative analysis result (Curvo et al., 2020). Images were converted to 8-bit with the detection threshold set between 0 and 50.

#### 2.2.3 Histological mapping onto µCT-scanned skulls

**FIGURE 2:**
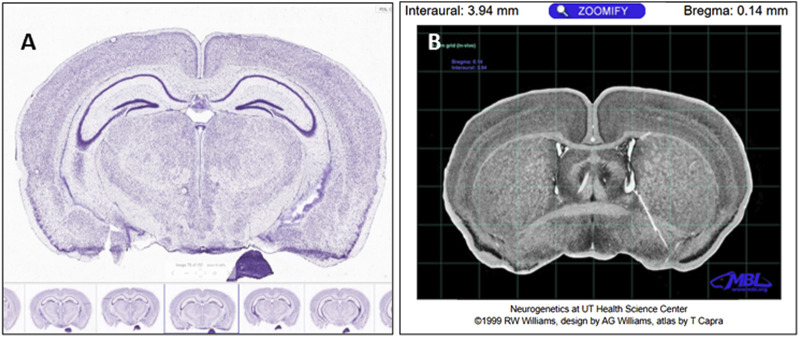
**(A)** Sagittal section number 78 on the Allen Brain Atlas. The Allen Brain Atlas mouse serial sections were used to identify neuroanatomical landmarks in our Von Kóssa-stained resin sections. **(B)** Upon identifying the location of the histology section using the Allen Brain Atlas, the Mouse Brain Library Atlas was used to identify the interaural distance and distance to the *bregma* of that area of the brain. Our Von Kóssa-stained resin sections could then be mapped onto the µCT scans, and the histological section could be considered in the context of the whole skull and calvarial defect (Allen Institute for Brain Science, 2004)

Von Kóssa-stained whole skull methyl methacrylate-embedded sections were mapped onto µCT scans of the same animal to validate areas/structures of *de novo* bone formation. First, a low-magnification micrograph of the entire histology section was compared to mouse brain sections from the Allen Mouse Brain Atlas (Allen Institute for Brain Science, 2004) (Figure 2A). The left/right lateral and superior/inferior portions of the brain visible on the histology sections were matched to brain sections in the Allen Mouse Brain Atlas. The stereotaxic location of each section was then identified using the Mouse Brain Library Atlas (Franklin and Paxinos, 1997) (Figure 2B). Using these stereotaxic coordinates, sections were located on the mouse’s µCT skull scan using bregma as a landmark. This enabled matching features from the histology scans and features on the µCT scans.

#### 2.2.4 Statistical evaluation

Both the quantified µCT and histomorphometric data were analyzed using GraphPad Prism version 10.0.2 (GraphPad Software, San Diego, California, United States). The normality of the distribution for each group was confirmed with a Shapiro-Wilk test, which yielded non-significant results (*p* > 0.05), suggesting no deviation from a normal distribution. Based on these findings, parametric statistical methods were deemed appropriate. Comparisons between the means of treatment groups—Control PLGA, PLGA 50 μm, PLGA 100 μm, and PLGA 200 µm—were performed using an unpaired two-tailed t-test. Homogeneity of variances was assumed. Differences were considered statistically significant at *p* < 0.05. The results are presented as boxplots depicting the median, interquartile ranges, and individual data points. Significant differences are indicated by asterisks (* where *p* < 0.05, ** where *p* < 0.01).

## 3 Results

### 3.1 Morphology of regenerative features observed by PLGA microsphere scaffolds implanted in calvarial bone defects

**FIGURE 3:**
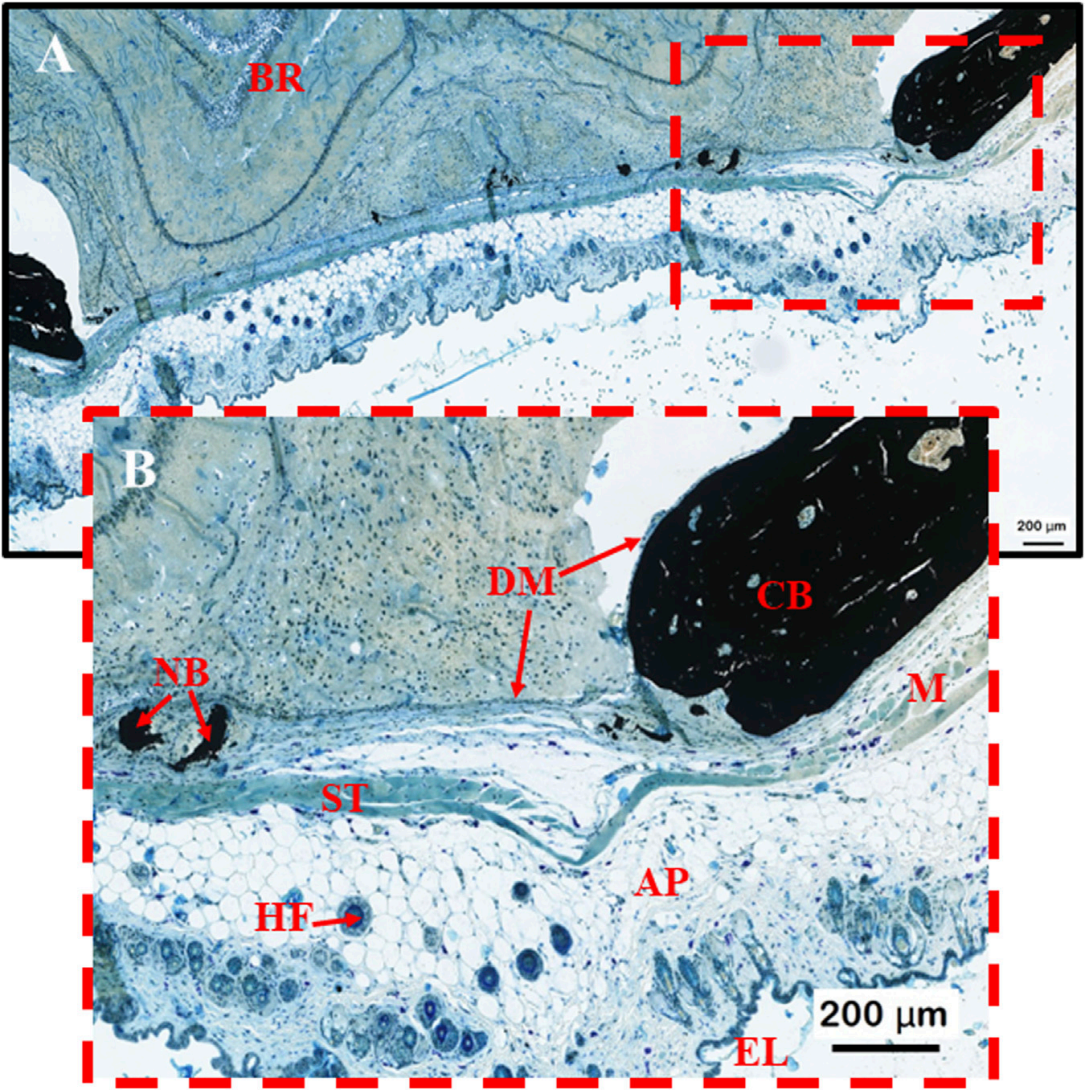
**(A, B)** Morphological control sample to help map important structures observed during the induction of bone formation by PLGA microsphere scaffolds within calvarial bone defect stained with von Kóssa. BR = brain; NB = new bone formation; ST = scar tissue (connective tissue formed from degenerated muscle; HF = hair follicle; DM = dura mater; CB = Calvarial bone; M = muscle tissue; AP = Adipose/Fat tissue; EL = epithelial skin layer.

**FIGURE 4:**
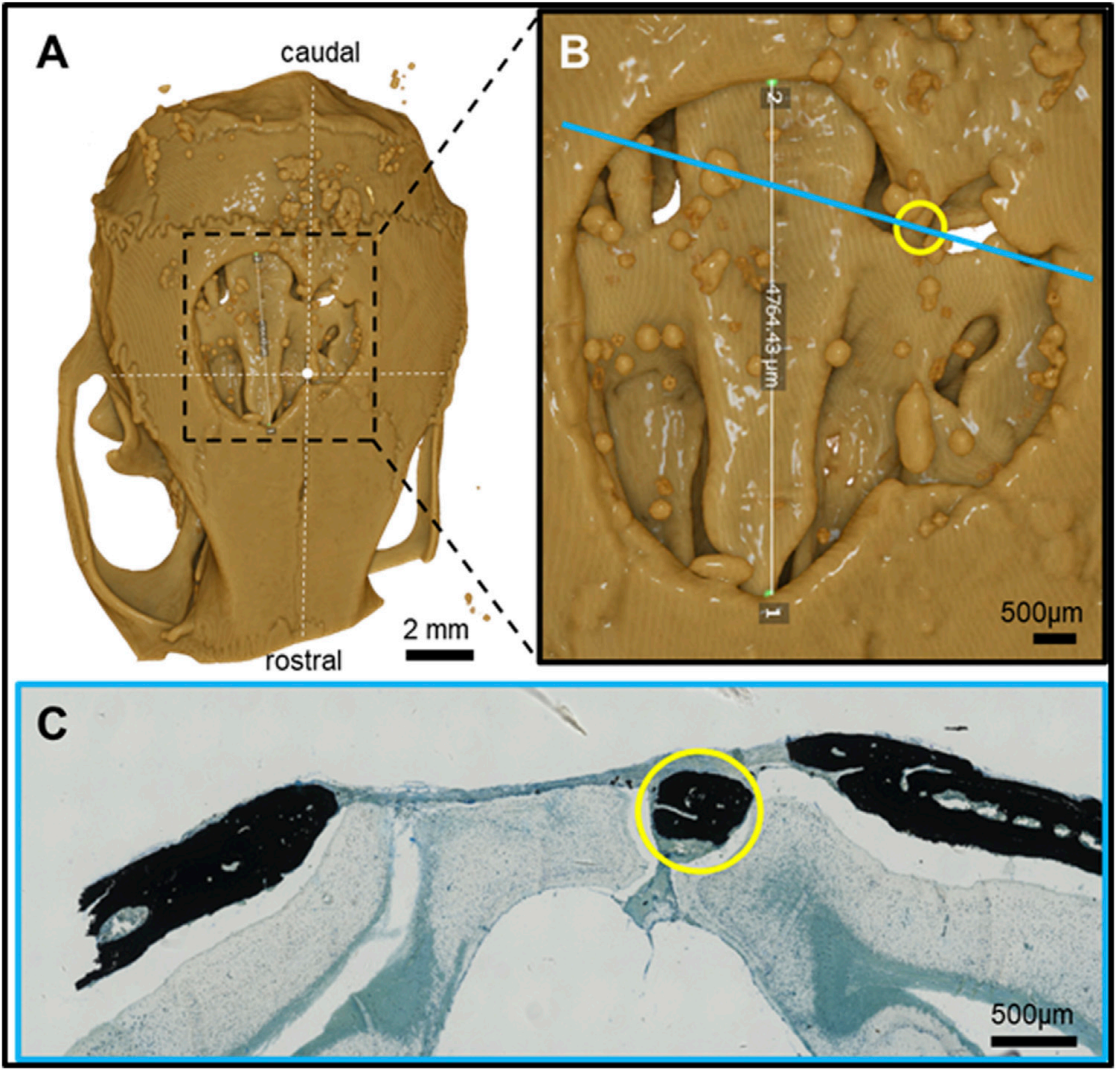
Feature mapping of histological section(s) stained with von Kóssa to µCT scanned images of calvarial defects implanted with pure PLGA control (no microspheres) scaffolds for 60 days. **(A)** µCT image (top view), **(B)** magnified section from A), and **(C)** histological slide located using the Allen Mouse Brain Atlas and position marked with blue line in B). Yellow circle = landmark structures correlated between histological sections and µCT.

**FIGURE 5:**
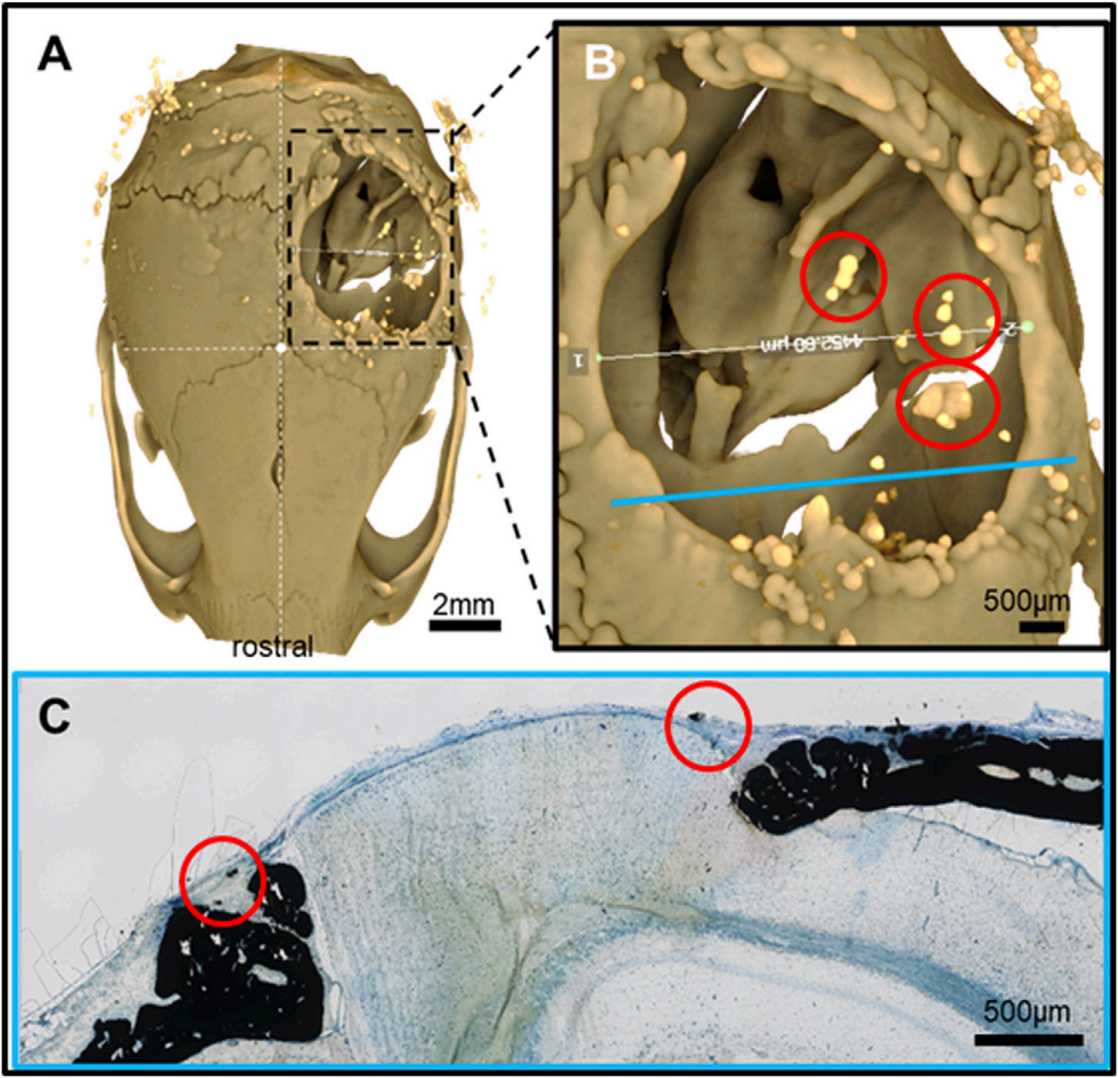
Feature mapping of histological section(s) stained with von Kóssa to μCT scanned images of calvarial defects with implanted PLGA microsphere scaffolds consisting of 50 μm microspheres and implanted for 60 days. **(A)** µCT image (top view), **(B)** magnified section from A), and **(C)** histological slide located using the Allen Mouse Brain Atlas and position marked with blue line in B). Red circle = osteoinduction; blue line = section position in the skull; diverse colored circles = landmark structures correlated between histological sections and µCT.

**FIGURE 6:**
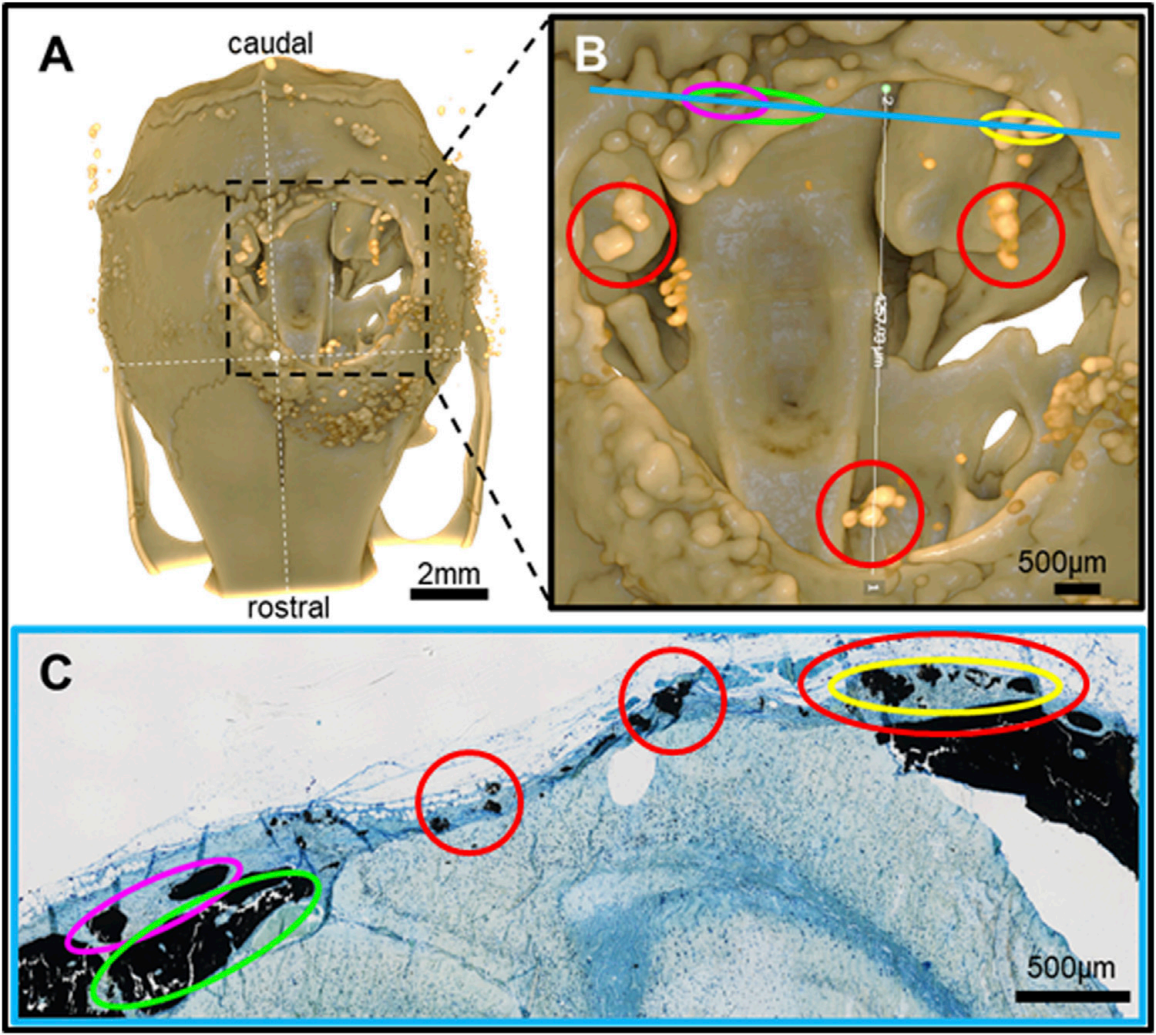
Feature mapping of histological section(s) stained with von Kóssa to μCT scanned images of calvarial defects with implanted PLGA microsphere scaffolds consisting of 100 μm microspheres and implanted for 60 days. **(A)** μCT image (top view), **(B)** magnified section from A), and **(C)** histological slide located using the Allen Mouse Brain Atlas and position marked with blue line in B). Red circle = osteoinduction; blue line = section position in the skull; diverse colored circles = landmark structures correlated between histological sections and μCT.

**FIGURE 7:**
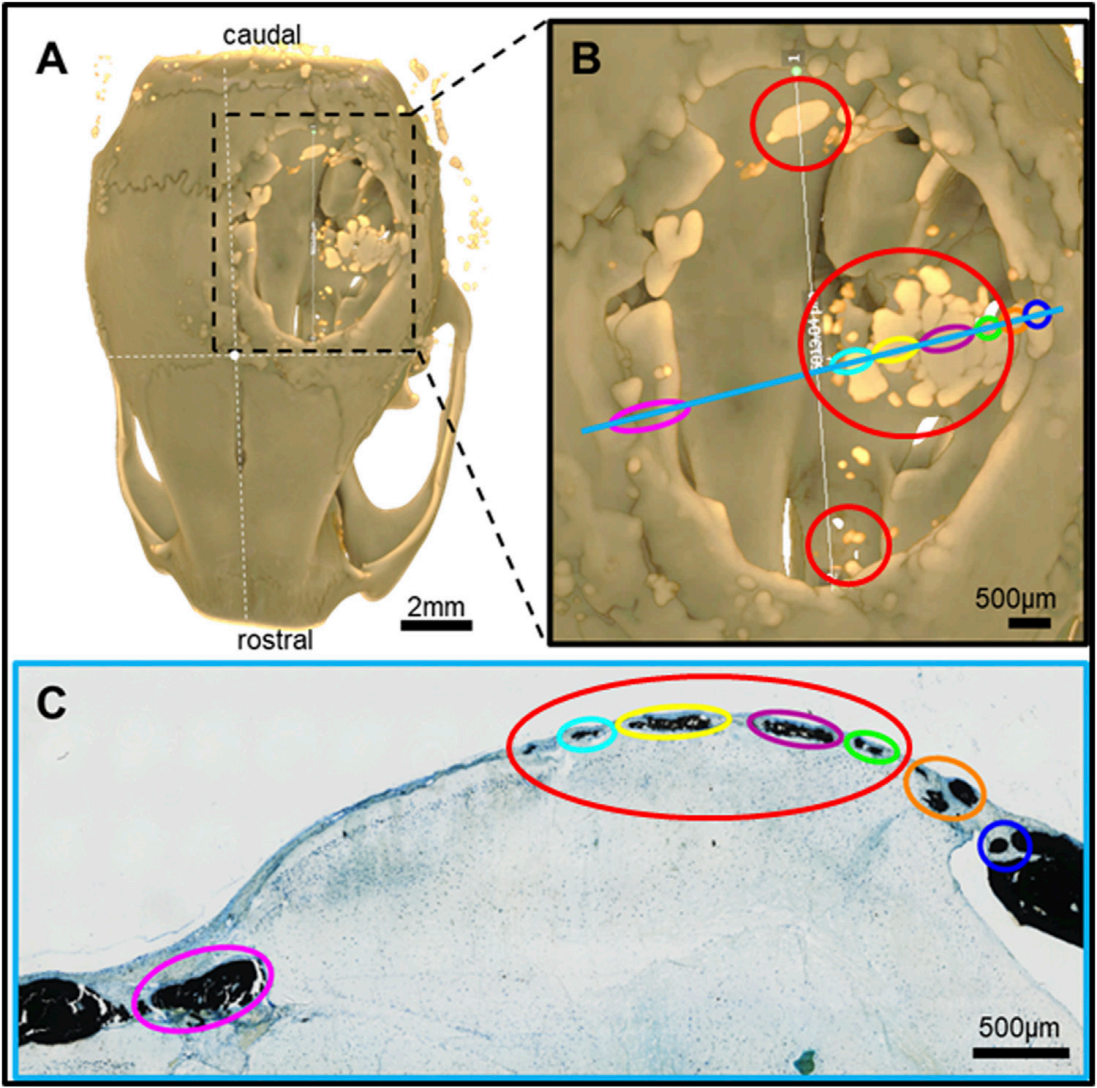
Feature mapping of histological section(s) stained with von Kóssa to μCT scanned images of calvarial defects with implanted PLGA microsphere scaffolds consisting of 200 µm microspheres and implanted for 60 days. **(A)** µCT image (top view), **(B)** magnified section from A), and **(C)** histological slide located using the Allen Mouse Brain Atlas and position marked with blue line in B). Red circle = osteoinduction; blue line = section position in the skull; diverse colored circles = landmark structures correlated between histological sections and µCT.

PLGA microsphere scaffolds implanted for 60 days in calvarial critical-size defects above the dura mater had degraded and been metabolized by the surrounding tissue (Figures 3A, B). Peripheral cranial tissue showed typical regeneration in the defect area across all groups. A specimen in which endothelial tissue and skin structures were still intact was chosen to properly verify and highlight unique morphological features, filtering out unwanted artifacts. The *dura mater* remained intact after 60 days, with scar tissue readily visible throughout specimens (Figures 3B, 4C, 5C, 6C, 7C). The endothelial layer with adipose tissue, hair follicles, and other structures was removed throughout specimens to ensure proper structural verification and new bone formation by the PLGA scaffolds (Figure 3B). The induction of bone formation was observed as black structures, via the von Kóssa stain, situated between the dura mater yet below the healing scar tissue zone (Figure 3B).

### 3.2 Morphological mapping of tissue repair features between histological and µCT scans of PLGA microsphere tissue repair

Pure PLGA-printed scaffolds showed only limited osteoconduction and no osteoinduction (Figure 4A; Figures 5A, B). The osteoconduction was observed mainly at the peripheral perimeter. The bone usually goes from a vertical cut surface to a more rounded triangle-like structure. This was the only bone formation in the control specimens that could be detected (Figures 4A, B). PLGA scaffolds constructed out of 50, 100, and 200 µm microspheres and implanted in cranial defects showed similar osteoconductive peripheral healing as the controls. However, unlike the controls, the 50, 100, and 200 µm microsphere-based scaffolds also showed osteoinduction (Figures 5–7; red circles). Osteoinduction was observed as the formation of small to large black stained areas, with osteoblast attachment on their surface, and situated between the dura mater and peri- and endocranial tissue layers (Figure 5C; Figure 6C; Figure 7C; red circles). In the µCT, these osteoinductive bone features were observed as dense roundish pockets within the open defect area (Figure 5B; Figure 6B; Figure 7B; red circles;; Supplementary Figure 1).

**FIGURE 8:**
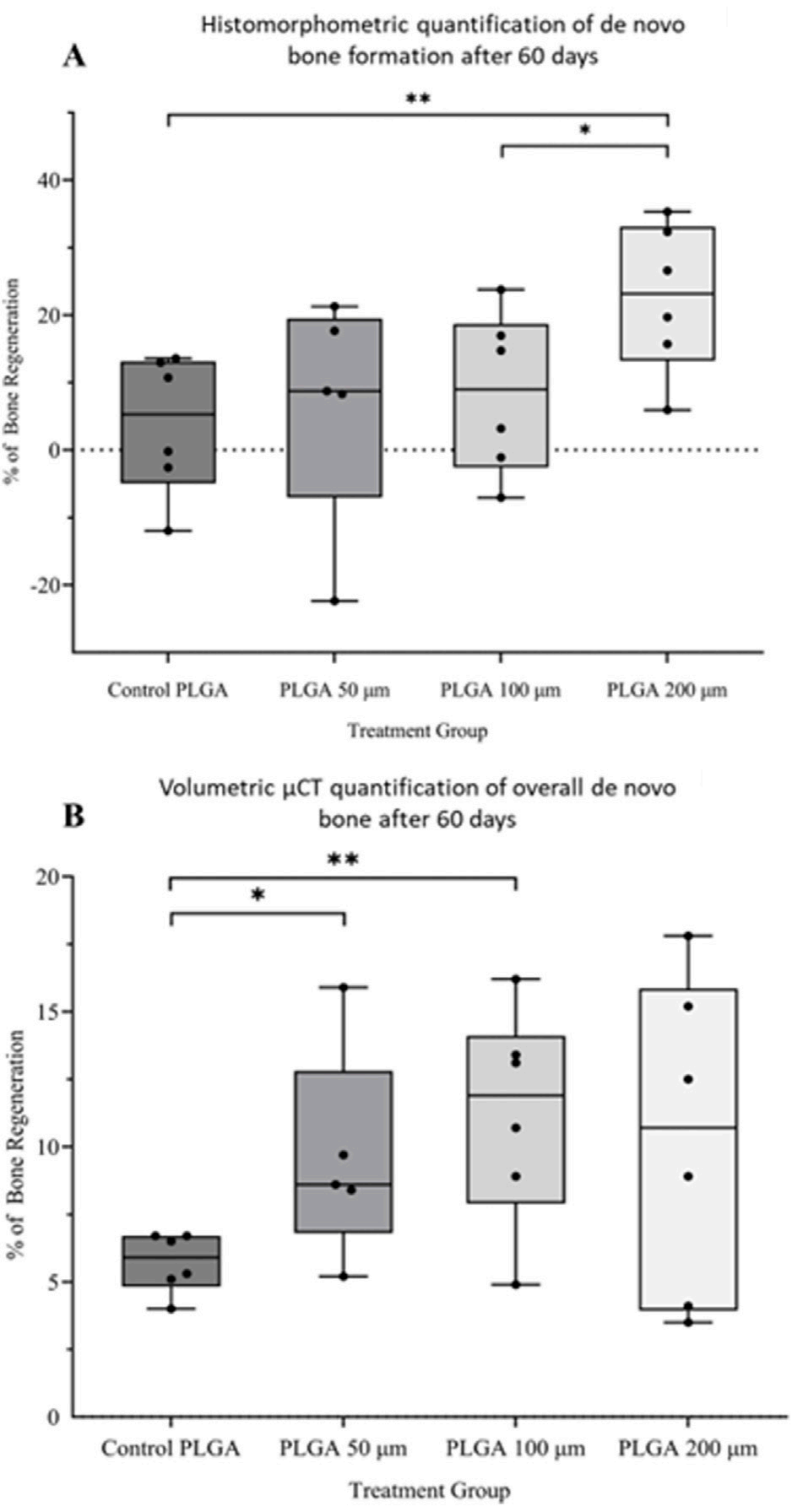
**(A)** Histomorphometric and **(B)** volumetric μCT quantification of new bone formation after 60 days in vivo by PLGA microsphere scaffolds. Pure PLGA scaffold is the control. *p < 0.05; **p < 0.01.

Histomorphometry and quantitative µCT analysis showed that the larger the microspheres became, the more bone formation was observed. Microsphere-based scaffolds constructed out of 50–100 µm diameter microspheres showed little difference compared to the control samples (Figures 8A, B). Only the scaffolds constructed out of 200 µm microspheres showed a significant increase in new bone formation after 60 days *in vivo* (Figures 8A, B) compared to all implantation groups.

## 4 Discussion

Surface characteristics, shape geometries, and material stiffness are defining factors essential for pre-committing stem cells to differentiate or transform into a specific cell type before any soluble molecular signal(s) have interacted with them (Dong et al., 2019; Gjorevski et al., 2022; Kuboki et al., 1998; von Erlach et al., 2018). For example, it has been shown that relatively soft matrices will more readily be favored for neurogenesis and the development of other soft tissue types, whereas comparatively rigid surfaces favor the hardier tissue formation processes like osteogenesis or certain chondrogenesis pathways (Dong et al., 2019; Gjorevski et al., 2022; von Erlach et al., 2018). Alternatively, osteoclasts favor a rough surface topography to differentiate properly, whereas osteoblasts require a smoother concave shape (Gupta et al., 2023). In the end, the soluble molecular signal, whatever it may be, is only meant to assist in committing the reaction down a specific developmental path. It is, therefore, imperative to design scaffolds that possess unique intrinsic capabilities that spontaneously guide cell differentiation through their specific architecture, providing a potent tool through which it is quite plausible that growth factors or the soluble molecular signal may not at all be relevant for new tissue development, as nature has revealed to us previously (Klar et al., 2013; Ripamonti, 1991; Ripamonti et al., 1999).

Research has shown that the sphere can perform multiple functions that primarily improve a scaffold’s intrinsic and unique topographical geometries. While any particle shape can carry soluble molecular signals that can substantially improve the temporal modulation of new tissue reformation (Yang and Zeng, 2019), the shape of the microsphere is its true benefit as it facilitates even degradation and the controllable release of encapsulated matter, allowing for the accurate prediction of biomolecule effects *in vivo*. Indeed, the shape of a sphere can impact the tissue formation response (Yang and Zeng, 2019). Stem cells cultured in monolayer do not differentiate as effectively as those cultured in a 3D structure because the sphere possesses the correct 3D configuration that cells use *in vivo*. Additionally, a sphere is the lowest energy configuration for a bound system, meaning cells function more efficiently to form new structures in and around a sphere than on a plain 2D surface. Most prominently, (Kuboki et al., 1998; Reddi and Huggins, 1973), and later (Ripamonti et al., 1999) were the first to show the power of the sphere/circle and its effect on spontaneous induction of bone formation in the heterotopic model. The “geometric bone-inductive concavity,” as (Ripamonti et al., 1999) called it, was later validated by (Rumpler et al., 2008) mathematically, determining that the curvature of a sphere drives new tissue growth and is directly proportional to the local curvature. While hydroxyapatite was used to maintain cellular specificity towards an osteogenic response, (Kuboki et al., 1998), pre-established that with just plain curvature, superior tissue formation was achievable, with different cells preferring different curvature shapes, concavity versus convexity for their tissue formation purposes. Based upon these previous findings, it can be suggested that the results in the present study regarding osteoinduction are indeed a “spontaneous” bone formation process linked to the shape characteristics of the microsphere size rather than the actual scaffold shape. The larger PLGA microsphere scaffolds created a more appropriate shape in the micro- and macroporous superstructure, significantly enhancing cellular differentiation and proliferation potential and leading to better healing and bone formation.

Pore sizes are known to affect cellular behavior (Han et al., 2021; Lu et al., 2022; Ma et al., 2022; Shi et al., 2021). Whether cellular migration, differentiation, proliferation, or subsequent matrix deposition, micro-and macropores are a geometric conductive force of new tissue formation. A pore size too small will allow only certain cell types to migrate and differentiate. In contrast, if the pore size is too large, cells will not do anything but rest patiently in a quiescent state, waiting for the appropriate stimulus (Lu et al., 2022). Indeed, it is known that macropores with an average pore size >50 μm are sufficient to allow for cellular proliferation and scaffold migration/colonization (Han et al., 2021; Lu et al., 2022). On the other hand, micropores <50 μm allow for better protein absorption, released by active cells, creating the relevant differentiation or transformation milieu in which cells can take on the relevant phenotype that is required to help in the formation of the sought-after tissue type (Han et al., 2021; Lu et al., 2022). It is, hence, plausible to suggest that the porosity level of the 50 µm microsphere scaffolds was sufficient to promote some cellular proliferation and, in turn, bone reformation but ultimately was insufficient to achieve the level of new tissue formation achieved by the larger microspheres. The larger the microspheres, the greater the porosity and, therefore, the superior the tissue regeneration. In future studies, knowing the exact cut-off size for the microsphere size and which microsphere sizes benefit which tissue formation lineage would be interesting. Theoretically speaking, if it can be determined that microspheres larger than 200 μm better promote bone formation, and in contrast, microspheres smaller than 50 μm are more suitable for neurological tissues, it would significantly streamline production methods, leading to substantially faster healing in patients. However, one part of the present study’s results is curious. It is well known that bone formation by induction does not just require the appropriate geometry and surface characteristics but also requires at least the presence of hydroxyapatite to help foster proper osteoinduction (Habibovic et al., 2005; Klar et al., 2013). The results show clear intramembranous ossification in both peripheral and central areas, with osteoconduction and osteoinduction, respectively. Osteoconduction is supported by the scientific literature regarding the closure of critical-sized defects in calvarial wounds (Bellido and Plotkin, 2011; Kalfas, 2001). Calvarial bone self-healing capabilities here require a guiding scaffold to help direct the peripheral bone healing front from properly causing defect wound closure (Kalfas, 2001). Specific to critical-sized defects in the calvarium, the dura mater also produces a specific concentration gradient of stimulatory and inhibitory signals (Benson et al., 2012; Lee et al., 2006; Ogle et al., 2004) that, with time, accumulate within the central region of the defect (He et al., 2010). This typically results in a donut shape of new bone formation, in which a central region does not support bone healing because of establishing an inhibitory bone formation zone caused by the dura mater (Jeong et al., 2015). As for the observed “spontaneous” osteoinductive healing reaction via the scaffold, regardless of the microsphere sizes analyzed, this could be possibly attributed to the PLGA, the microporosity, and their behavior under *in vivo* conditions. Future studies must investigate this further and determine if there is a direct correlation between microspheres and PLGA degradation periods on the spontaneous induction of bone formation and if this is translatable to other synthetic polymer microspheres. Such information would be clinically invaluable, allowing for superior control of the tissue regenerative processes.

Regarding the limitations of the present study, the single-time data collection point of 60 days in the mice does not provide sufficient temporal tissue developmental transition information. Additionally, the size differences between control scaffolds versus microsphere scaffolds could have affected the regeneration process within the controls. The present pilot study helped to determine how the research direction should evolve based upon discoveries. Future studies need to include standardized printing parameters between controls and actual samples including the inclusion of more data collection points during the animal study. This would allow for better tracking the healing process transitions, thereby enabling a better classification of the tissue processes. Additionally, molecular gene and protein expression data would have provided better answers and strengthened histomorphometric observations. Finally, the study lacked the use of other scaffolds, known to be spontaneously inductive, such as coral-derived matrices (Ripamonti, 1991), including pre-analytical *in vitro* cell culture data to which PLGA microspheres could have been compared and to determine how microsphere scaffolds are affecting cellular behavior. Through future studies, these limitations will be gradually incorporated into more detailed experiments that, with time, should generate superior scaffolds comprised of microspheres and entrapped modulatory biomolecules that finally and adequately recapitulate proper autogenous bone regeneration.

## 5 Conclusion

Microsphere-based scaffolds show much promise for becoming an excellent and safe clinical option replacing the autogenous bone graft golden standard. From the present study, PLGA microsphere scaffolds possess unique properties that support bone regeneration by supporting osteoconduction and stimulating, in our opinion, true spontaneous osteoinduction. The study demonstrated that PLGA microsphere-based scaffolds promote bone growth even in the absence of additional factors to promote osteogenesis. The quality, quantity, and exact localization of cranial bone growth in critical size defects in mice remains to be fully characterized. Future studies will explore not only scaffold characteristics such as microsphere size but also the osteogenic impacts of microsphere-encapsulated biomolecules to create new medical innovative solutions that have the potential to be used clinically in the future to promote new bone growth for defects in multiple bones for cranial defects of varying shapes and sizes, and across different age groups.

## Data Availability

The raw data supporting the conclusions of this article will be made available by the authors, without undue reservation.
