# Supplementary material for: The induction of bone formation by 3D-printed PLGA microsphere scaffolds in a calvarial orthotopic mouse model: a pilot study
Source: Front Bioeng Biotechnol. 2024 Oct 25;12:1425469. doi: 10.3389/fbioe.2024.1425469 (PMC11544432; doi:10.3389/fbioe.2024.1425469)
Supplement: Supplementary file 1 [file Image1.pdf]

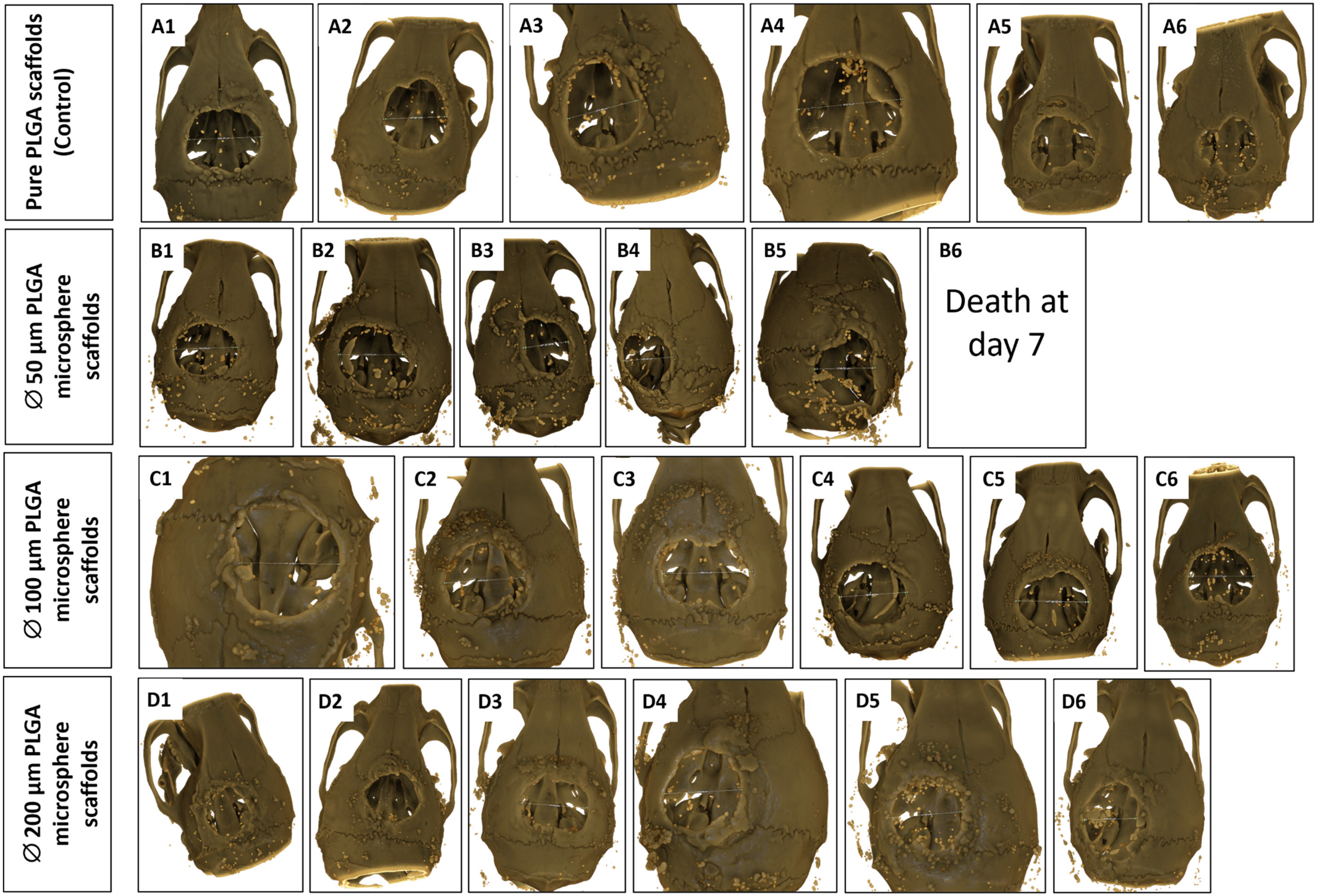

Figure S-1. (A1-A6) µCT scans showing the distribution of new bone formation at day 60 in murine calvarial defects implanted with pure PLGA scaffolds; **(B1-B6)** µCT scans showing the distribution of new bone formation at day 60 in murine calvarial defects implanted with scaffolds consisting of ø 50µm PLGA microspheres; **(C1-C6)** µCT scans showing the distribution of new bone formation at day 60 in murine calvarial defects implanted with scaffolds consisting of ø 100µm PLGA microspheres; **(D1-D6)** µCT scans showing the distribution of new bone formation at day 60 in murine calvarial defects implanted with scaffolds consisting of ø 200µm PLGA microspheres.
